# Observations on the Use of the Carbonate of Iron in the Treatment of Tic Douloureux; with the History of a Case Illustrative of the Efficacy of the Remedy

**Published:** 1821-09

**Authors:** Benjamin Hutchinson

**Affiliations:** Member of the Royal College of Surgeons in London.


*Observations on the Use of the Carbonate of Iron in the Treatment of Tic Douloureux; with the History of a Case illustrative of the Efficacy of the Remedy.* By BENJAMIN HUTCHINSON, Member of the Royal College of Surgeons in London. [In a Letter to the Editor.]

THROUGH the medium of your Journal, I hope you will allow me to mention to my medical brethren my intention of submitting to their consideration, in a short time, a second edition of my little pamphlet on the Treatment of Tic Douloureux; and I feel impelled, by a variety of motives, to request the indulgence of a very short digression from the immediate subject of this communication. The kind and very flattering terms in which my little work has been noticed by yourself, in the first place, and by other able and respectable medical and literary reviewers, have been a source of truly gratifying sensations. It has fortunately escaped the censure of all who have taken the trouble to analyze it, and has received the praise and approbation of that class of men whose good and favourable opinions are really estimable. The French reviewers, with their wonted complaisance, have dealt out their encomiums with too lavish a hand; and, to the concluding remark of a critique in a Number of the "Journal Général de Médecine," I have some anxiety to make a reply. This reviewer thinks it would have been more satisfactory had I given a history of my unsuccessful as well as of my more fortunate cases, as he (the reviewer) cannot imagine that I have been uniformly successful in the treatment of this obstinate disease. Had I advanced the bold and untenable opinion, that the carbonate of iron acted as a never-failing specific in this disease,—that it never disappointed us in curing the most obstinate case of tic douloureux,—or that the malady of thirty years' continuance was equally amenable to its influence as that of only three weeks or three months' duration, I might probably have merited this slight reproof. A professional experience of nearly thirty years, however, has taught me not to place implicit confidence in the curative and specific agency of any remedy at present known. We are not in these days to be taught, that the bark will not always subdue the paroxysms of intermittent fever, and that even mercury* has been known to fall short of the subjugation of syphilis. The carbonate of iron has, in a few instances under my own cognizance, been inadequate to the difficult task of curing the tic douloureux. I can however

* "The fact that it [mercury] acts with equal rapidity and effect upon all cases of inflamed iris, whatever their origin, seems to me to show that the idea of a *specific* antisyphilitic virtue possessed by this mineral is an erroneous one."—*Vide* COOPER's *and* TRAVERS' *Surgical Essays*, Part I. 3d edition.

assert, that, in these very few instances, it has done more towards alleviation than any other known remedy. The carbonate of iron has been frequently given in very improper cases, and in the most injudicious manner, without any due consideration of peculiarities of constitution, or of the proximate cause of the disease; circumstances essentially requisite to be attended to in the administration of a powerfully stimulant remedy. In the very few unsuccessful cases under my own management, I can almost uniformly attribute the want of success rather to the absence of a due perseverance in the use of the remedy, than to any failure in its power. It ought also to be perfectly understood that collateral means of relief have uniformly been used in conjunction with the iron, and that the treatment is by no means confined to one remedy. Where symptoms of active inflammation have existed, the means usually employed to subdue inflammation have been had recourse to, and the iron either suspended or never used.

I have of late been consulted by many persons under the injudicious use of iron, when high inflammatory action has existed in the nerves particularly affected, and when the neighbouring parts and the general constitution have sympathized with the augmented arterial impulse. To the intelligent readers of your Journal it is needless for me to explain the results of a practice founded on a complete ignorance of all medical principles. Mr. Abernethy very justly remarks, that "nerves strikingly resemble arteries in their modes of communication: sometimes they conjoin even by considerable branches, such as must be manifest in common dissections; but they communicate in surprising numbers by their minute ramifications. This circumstance is not, perhaps, so familiarly known to professional men, since it cannot be perceived unless in the course of a very minute dissection; and, to understand how numerous these communications are, the representations given by the German authors of their delicate and laborious dissections may be advantageously consulted." It is my intention to prefix to a second edition of my little pamphlet a well-executed plate, showing the distribution of the nerves principally affected by tic douloureux.

You will now, I hope, allow me to conclude this letter by the narration of a case of tic douloureux, recently cured by the means recommended in my little pamphlet.

Mr. Todd, a very respectable farmer and grazier, residing at Farnsfield in the county of Nottingham, in the twenty-sixth year of his age, of sober habits, and of a delicate lax fibre, began to complain of uneasy sensations in the left side of his face, about a year and a half since, and which he at first attributed to carious teeth. The pain extended to the left ala nasi, the upper lip, the superior bicusped teeth, the temple, the upper and lower lip. The infra-orbitar nerve, with its manifold ramifications, appeared to be the principal seat of the disease, which consisted of paroxysms, in the periods of recurrence, in degree and duration, extremely irregular. They would at times excite only moderate complainings, but more generally the most violent screaming and contortions. The pain would sometimes begin below the eye, and descend to the upper and lower lip. The continuance of these fits of pain was very uncertain, frequently remaining five minutes, and sometimes only a few seconds. A perfect freedom from pain and extreme good health were enjoyed in the intervals of the disease. The scalding, burning, and tearing and boring sensations, gave my patient the power of describing with accuracy the kind of torture with which he was afflicted. There did not appear to be any period of the day in which these paroxysms were more violent, or their recurrence more frequent. His nights were bad, and the intervals of ease were generally spent in unrefreshing slumbers, alarmed by dreams excited by suggestions of approaching attacks. The paroxysms appeared to come on spontaneously, no external cause producing them; neither did the actions of mastication or of speaking excite this effect. The weaker attacks would frequently admit of alleviation from a compression of the infra-orbitar branches, about an inch and a half below the eye. The parts affected always retained their natural appearance, notwithstanding the patient himself always complained of a sense of tumefaction. This train of symptoms clearly pointed out the character of the disease: the outlines of the case were so strongly marked, that the suggestion of any other malady than the one now treated of was wholly precluded. The plan of cure appeared to my mind equally obvious. Under the mistaken idea that the disease originated in my patient's teeth, he suffered the extraction of three molares, with the painful mortification of not receiving the most trivial relief: on the contrary, an increase of all his tortures immediately succeeded; the violence committed in the neighbourhood of the distracted nerves seemed to aggravate their state of irritability in a tenfold degree, and his miseries appeared to admit of no alleviation. His complaint was now treated as a rheumatic affection, and various internal and external means were tried, with the same unavailing effects. At the expiration of a years' suffering, Mr. Todd applied to me; and I immediately commenced that plan of management which experience had taught me to be the most successful. In the middle of last May, he began to take one drachm of the carbonate of iron three times a-day; and I directed him to make use of an ointment to the affected cheek every night, consisting of the tartrite of antimony, powdered opium, camphor, and mercurial ointment; one drachm of which was used each evening, until a plentiful crop of pustules appeared: its use was then suspended a few days, and renewed when the eruptions were healed.

This plan was duly persevered in for three weeks, when my patient had experienced some very trifling degree of relief. The medicine now produced uneasy sensations in his bowels, which were removed, and in future prevented, by mixing the powder with the confect. aromatic. instead of honey; and, if diarrhœa at any time accompanied its exhibition, five drops of the tinct. opii added to each dose restrained any inordinate action of this nature.

During the ensuing six weeks, my patient pretty regularly persevered in the same plan, modifying and altering the treatment according to existing symptoms, occasionally omitting the medicine a day or two from unforeseen circumstances. During this period he gradually lost his pains, and he is now perfectly well.

*Southwell, Notts.; August* 1, 1821.

